# Contribution of *PNPLA3* gene polymorphisms to hepatocellular carcinoma susceptibility in the Chinese Han population

**DOI:** 10.1186/s12920-022-01394-7

**Published:** 2022-11-29

**Authors:** Dongwei Gong, Shizong Li, Zhiwei Yu, Kaiqiong Wang, Xin Qiao, Changxiong Wu

**Affiliations:** Hepatopancreatobiliary Surgery, Hainan General Hospital, Hainan Affiliated Hospital of Hainan Medical University, #19, Xiuhua Road, Xiuying District, Haikou City, Hainan Province China

**Keywords:** *PNPLA3*, Polymorphism, Hepatocellular carcinoma, Susceptibility, Chinese Han population

## Abstract

**Objectives:**

The purpose of this study was to investigate the association of *PNPLA3* single nucleotide polymorphisms (SNPs) (rs738409 C > G, rs3747207 G > A, rs4823173 G > A, and rs2896019 T > G) with hepatocellular carcinoma (HCC) susceptibility.

**Methods:**

This case–control study included 484 HCC patients and 487 controls. Logistic regression analysis was performed to study the associations of *PNPLA3* gene polymorphisms with HCC susceptibility, and odds ratios with their corresponding 95% confidence intervals were calculated to evaluate these correlations.

**Results:**

In the overall analysis, we found that the G allele (OR = 1.25, 95% CI = 1.04–1.50, *p* = 0.018, false discovery rate (FDR)-*p* = 0.035) and GG genotype (OR = 1.59, 95% CI = 1.06–2.39, *p* = 0.024, FDR-*p* = 0.048) of rs2896019 were significantly associated with increased HCC susceptibility. In stratified analysis, we found that all four SNPs were related to increased HCC susceptibility in subjects aged > 55 years. In haplotype analysis, the GAAG haplotype was significantly associated with increased HCC susceptibility (OR = 1.25, 95% CI = 1.03–1.53, *p* = 0.023, FDR-*p* = 0.046). Besides, we noticed that rs738409 was significantly correlated with alpha-fetoprotein (AFP) (*p* = 0.007), and HCC patients with the GG genotype had a higher level of AFP.

**Conclusions:**

Our study suggested that *PNPLA3*-rs2896019 was significantly associated with an increased susceptibility to HCC.

**Supplementary Information:**

The online version contains supplementary material available at 10.1186/s12920-022-01394-7.

## Introduction

Primary liver cancer is currently the fourth most common malignancy and the second leading cause of cancer-related death in China, seriously threatening people’s life and health [1]. Hepatocellular carcinoma (HCC) is the most common form of primary liver cancers, accounting for approximately 75–85% of primary liver cancers [2]. The early symptoms of HCC are not obvious, and the disease will not be detected until it develops to a certain stage. Modern medical technology can treat HCC in a timely and effective manner and prolong patients’ life to a certain extent. Unfortunately, HCC cannot be completely cured. Additionally, it is prone to recurrence and metastasis, and has a poor prognosis [3]. HCC is a disease with multifactorial etiologies, including viral factors, such as hepatitis B virus (HBV) and hepatitis C virus (HCV), and non-viral factors, such as smoking, alcohol consumption, aflatoxin, and obesity [4]. Remarkably, these risk factors are not entirely responsible for the rising morbidity and mortality of HCC. With the rapid development of molecular genetics, researchers have found that genetic mutations, such as single nucleotide polymorphisms (SNPs), have become one of the important basic reasons for the occurrence of HCC [5]. Hence, it is urgent to study the relationship between SNPs and HCC susceptibility, which can provide theoretical guidance for the clinical diagnosis and prevention of HCC.

Patatin-like phospholipase domain-containing 3 gene (*PNPLA3*), also known as adiponectin, encodes a transmembrane protein composed of 481 amino acids and is expressed on the hepatocyte membrane, which can regulate lipid metabolism, inflammatory mediators and so on [6, 7]. *PNPLA3*, located on the long arms (q) (22q.13.31) of human chromosome 22, belongs to the patatin-like phospholipase family and is highly expressed in liver and fat [8, 9]. At present, numerous studies are concerned with the association of *PNPLA3* gene polymorphisms with non-alcoholic fatty liver disease (NAFLD) [10–12], alcoholic liver disease (ALD) [13], liver fibrosis (LF) [14, 15], and liver cirrhosis (LC) [16]. On the contrary, very little pays attention to the association of *PNPLA3* gene polymorphisms with HCC. Therefore, further research on the relationship between *PNPLA3* gene polymorphisms and HCC susceptibility is of great value.

In this study, we performed association analyses to determine the effect of *PNPLA3* gene polymorphisms on HCC susceptibility in the Chinese Han population. Based on the 1000 Genomes Project database and Haploview software, combined with Hardy–Weinberg equilibrium (HWE) test and primer design principles, we selected four SNPs (rs738409, rs3747207, rs4823173, and rs2896019) of the *PNPLA3* gene for further study. Finally, we assessed the relationship between *PNPLA3* SNPs and HCC susceptibility, which can provide clues for identifying more genetic polymorphisms related to HCC susceptibility.

## Materials and methods

### Study subjects

Power analysis was carried out before the study to determine the required sample size. According to power analysis, the case group and control group should consist of at least 484 and 486 individuals, respectively. Accordingly, this study included 971 subjects (484 HCC patients and 487 controls) from Hainan General Hospital. All patients with HCC were diagnosed by imaging, hematological molecular and pathological examinations in accordance with the guidelines for the diagnosis and treatment of primary HCC in China. The control group was healthy people who underwent physical examination during the same period as cases. The inclusion criteria for the control group were the blood routine and biochemical indicators within a normal reference range, as well as no endocrine, cardiovascular, kidney, and liver diseases. All subjects were genetically unrelated Chinese Han people. This study was approved by the Ethics Committee of Hainan General Hospital, and all subjects signed the informed consent.

### SNP selection and genotyping

Four genetic polymorphisms (rs738409, rs3747207, rs4823173, and rs2896019) in *PNPLA3* were screened according to the following procedures. Firstly, all mutant loci in *PNPLA3* were downloaded from the 1000 Genomes Project. Secondly, Haploview software was utilized to filter SNPs, depending on these parameters: HWE > 0.01 and minor allele frequency (MAF) > 0.05. Finally, combined with primer design and literature search, four candidate SNPs were identified. Peripheral venous blood (5 mL) was drawn from each subject, placed in an EDTA anticoagulant tube and stored at 4 °C for DNA extraction. GoldMag extraction kit (GoldMag Co., Ltd., Xi’an, China) was used to extract genomic DNA from peripheral venous blood. DNA concentration was detected using NanoDrop 2000 (Thermo Scientific, Waltham, Massachusetts, USA). The primer design software Assay Designer 3.1 was applied to design primers for four SNPs (Additional file 1: Table S1). Genotyping of four SNPs was performed using the Agena MassARRAY system.

### Statistical analysis

The independent samples *t*-test (continuous variables) and chi-square test (categorical variables) were used to analyze sample characteristics. The HWE test for SNP genotypes in the control group was performed using the chi-square test. Logistic regression analysis was used to evaluate the association between *PNPLA3* gene polymorphisms and HCC susceptibility, and odds ratios (ORs) and 95% confidence intervals (CIs) were calculated to assess the influence of different alleles and genotypes on HCC susceptibility under multiple genetic models (co-dominant, dominant, recessive, and log-additive models). At the same time, PLINK software was applied to perform haplotype analysis. Multifactor dimensionality reduction (MDR) software 3.0.2 was used to determine the effect of SNP-SNP interactions on HCC susceptibility. To reduce false positives, a tenfold cross-validation procedure was used to generate data. The best model was selected based on the maximum cross-validation consistency (CVC) and testing balanced accuracy. Besides, false discovery rate (FDR) analysis was carried out to correct multiple testing, while false-positive report probability (FPRP) analysis was conducted to test whether significant results were credible. Finally, all statistical analyses were performed using SPSS 22.0, and *p* < 0.05 was considered statistically significant.

## Results

### Participant characteristics

The basic information about 484 HCC patients and 487 healthy controls is shown in Table 1. The average ages of HCC patients and controls were 55.06 ± 11.33 years and 55.07 ± 11.02 years, respectively, and no significant difference in age distribution was found between the two groups. In addition, there were also no significant differences in the distribution of gender, smoking and carbohydrate antigen 50 (CA 50) between the two groups. However, the differences in the levels of carcino-embryonic antigen (CEA) (*p* ˂ 0.001), alpha-fetoprotein (AFP) (*p* = 0.028), carbohydrate antigen 125 (CA 125) (*p ˂* 0.001), and carbohydrate antigen 199 (CA 199) (*p* ˂ 0.001) between the two groups were worthy of note.

**Table 1:** Basic characteristics of subjects

Parameter	Case (*n* = 484)	Control (*n* = 487)	*p*
Age (Years, Mean ± SD)	55.06 ± 11.33	55.07 ± 11.02	0.989^a^
> 55	231(47.7%)	226 (46.4%)	
≤ 55	253(52.3%)	261 (53.6%)	
*Gender, n (%)*			0.799^b^
Male	376(77.7%)	375 (77.0%)	
Female	108(22.3%)	112 (23.0%)	
*Smoking, n (%)*			0.051^b^
Yes	238(49.2%)	234 (48.0%)	
No	246(50.8%)	253 (52.0%)	
*Serum tumor marker levels ( Mean ± SE)*			
CEA (ng/mL)	14.79 ± 0.97	2.22 ± 0.07	**˂ 0.001**^**a**^
AFP (ng/mL)	279.26 ± 124.32	3.19 ± 0.11	**0.028**^**a**^
CA50 (U/mL)	8.33 ± 0.80	7.00 ± 0.29	0.118^a^
CA125 (U/mL)	115.05 ± 15.93	10.29 ± 0.29	**˂ 0.001**^**a**^
CA199 (U/mL)	54.39 ± 6.21	10.17 ± 0.40	**˂ 0.001**^**a**^

*CEA* carcino-embryonic antigen; *AFP* alpha-fetoprotein; *CA 50* carbohydrate antigen 50; *CA 125* carbohydrate antigen 125; *CA 199* carbohydrate antigen 199

*p*^a^-value was calculated by *t*-test

*p*^b^-value was calculated by χ^2^ test

Bold values indicate statistical significance

### Basic information and allele frequencies of *PNPLA3* SNPs

The basic information and allele frequency distribution of four *PNPLA3* SNPs (rs738409, rs3747207, rs4823173, and rs2896019) in cases and controls are shown in Table 2. These four SNPs in the control group were all in line with HWE (*p* > 0.05). The allele frequency distribution of rs738409 (*p* = 0.045), rs4823173 (*p* = 0.017) and rs2896019 (*p* = 0.018) was statistically different between the two groups. After FDR correction, the results indicated that the minor allele of rs2896019 was significantly associated with increased HCC susceptibility (FDR-*p* = 0.035).

**Table 2:** Basic information and allele frequencies of rs738409, rs3747207, rs4823173, and rs2896019 in *PNPLA3*

SNP_ID	Gene	Chr	Base pair	Allele	MAF		HWE	OR (95% CI)	*χ*^2^	*p*	FDR-*p*
					Case	Control	*p*-value				
rs738409	*PNPLA3*	22	43,928,847	C > G	0.422	0.378	0.209	1.21 (1.00–1.45)	4.029	**0.045**	0.060
rs3747207	*PNPLA3*	22	43,928,975	G > A	0.415	0.374	0.066	1.19 (0.99–1.43)	3.513	0.061	0.061
rs4823173	*PNPLA3*	22	43,932,850	G > A	0.420	0.367	0.095	1.25 (1.04–1.50)	5.693	**0.017**	0.068
rs2896019	*PNPLA3*	22	43,937,814	T > G	0.423	0.371	0.207	1.25 (1.04–1.50)	5.640	**0.018**	**0.035**

*SNP* single nucleotide polymorphism; *Chr* chromosome; *MAF* minor allele frequency; *HWE* Hardy–Weinberg equilibrium; *OR* odds ratio; *95% CI* 95% confidence interval; *χ*^*2*^ chi-square; *FDR* false discovery rate

Bold values indicate statistical significance

*p*-value was calculated by χ^2^ test

### Association between *PNPLA3* gene polymorphisms and HCC susceptibility

The overall analysis indicated that three SNPs were associated with increased susceptibility to HCC (Table 3). Specifically, rs738409 was significantly correlated with an increased susceptibility to HCC (homozygous model: OR = 1.49, 95% CI = 1.00–2.22, *p* = 0.049; log-additive model: OR = 1.22, 95% CI = 1.01–1.48, *p* = 0.038). Besides, rs4823173 was closely associated with increased susceptibility to HCC (homozygous model: OR = 1.63, 95% CI = 1.08–2.46, *p* = 0.019; dominant model: OR = 1.35, 95% CI = 1.04–1.77, *p* = 0.027; log-additive model: OR = 1.28, 95% CI = 1.06–1.55, *p* = 0.012). Rs2896019 was also linked to an increased susceptibility to HCC (heterozygous model: OR = 1.33, 95% CI = 1.01–1.76, *p* = 0.045; homozygous model: OR = 1.59, 95% CI = 1.06–2.39, *p* = 0.024; dominant model: OR = 1.38, 95% CI = 1.06–1.81, *p* = 0.018; log-additive model: OR = 1.28, 95% CI = 1.06–1.55, *p* = 0.012). After FDR correction, rs4823173 showed borderline association with increased HCC susceptibility in the log-additive model (FDR-*p* = 0.049), and rs2896019 was remarkably related to increased susceptibility to HCC in both homozygous (FDR-*p* = 0.048) and additive (FDR-*p* = 0.024) models.

**Table 3:** Association between *PNPLA3* polymorphisms and HCC susceptibility

SNP_ID	Model	Genotype	Case	Control	Adjusted		
					OR (95% CI)	*p*	FDR-*p*
rs738409	Co-dominant	CC	154	180	1		
		CG	250	240	1.22(0.92–1.62)	0.160	0.214
		GG	79	62	1.49(1.00–2.22)	**0.049**	0.065
	Dominant	CC	154	180	1		
		CG + GG	329	302	1.28(0.98–1.67)	0.072	0.096
	Recessive	CC + CG	404	420	1		
		GG	79	62	1.33(0.92–1.91)	0.128	0.128
	Log-additive	–	–	–	1.22(1.01–1.48)	**0.038**	0.051
rs3747207	Co-dominant	GG	156	181	1		
		GA	254	248	1.19(0.90–1.57)	0.216	0.216
		AA	74	242	1.48(0.99–2.23)	0.058	0.058
	Dominant	GG	156	181	1		
		GA + AA	328	306	1.25(0.96–1.63)	0.104	0.104
	Recessive	GG + GA	410	429	1		
		AA	74	58	1.34(0.92–1.94)	0.127	0.170
	Log-additive	–	–	–	1.21(1.00–1.47)	0.050	0.050
rs4823173	Co-dominant	GG	152	184	1		
		GA	256	242	1.28(0.97–1.70)	0.075	0.150
		AA	75	56	1.63(1.08–2.46)	**0.019**	0.078
	Dominant	GG	152	184	1		
		GA + AA	331	298	1.35(1.04–1.77)	**0.027**	0.053
	Recessive	GG + GA	408	426	1		
		AA	75	56	1.40(0.96–2.04)	0.077	0.308
	Log-additive	–	–	–	1.28(1.06–1.55)	**0.012**	**0.049**
rs2896019	Co-dominant	TT	150	186	1		
		TG	257	241	1.33(1.01–1.76)	**0.045**	0.181
		GG	76	60	1.59(1.06–2.39)	**0.024**	**0.048**
	Dominant	TT	150	186	1		
		TG + GG	333	301	1.38(1.06–1.81)	**0.018**	0.071
	Recessive	TT + TG	407	427	1		
		GG	76	60	1.34(0.93–1.94)	0.116	0.232
	Log-additive	–	–	–	1.28(1.06–1.55)	**0.012**	**0.024**

*HCC* hepatocellular carcinoma; *SNP* single nucleotide polymorphism; *OR* odds ratio; *95% CI* 95% confidence interval; *FDR* false discovery rate

*p*-value was calculated by logistic regression analysis with adjustments for age, gender and smoking

Bold values indicate statistical significance

### Stratified analysis of the association between *PNPLA3* gene polymorphisms and HCC susceptibility

Basic information including age, gender, and smoking status was collected from all subjects to perform stratified analyses. Age-stratified analysis showed that rs738409, rs3747207, rs4823173, and rs2896019 were all associated with increased HCC susceptibility in subjects aged > 55 years (Table 4). After FDR correction, these four SNPs were still strikingly related to increased HCC susceptibility in subjects aged > 55 years, suggesting that age differences might affect the relationship between *PNPLA3* gene polymorphisms and HCC susceptibility. Stratified analysis based on gender (Additional file 1: Table S2) and smoking status (Additional file 1: Table S3) showed that these four SNPs were all correlated with increased HCC susceptibility in men and women, as well as smokers and nonsmokers, which indicated that the effect of these SNPs on HCC susceptibility might not be related to gender and smoking status.

**Table 4:** Association between *PNPLA3* polymorphisms and HCC susceptibility stratified by age

SNP_ID	Model	Genotype	Age > 55					Age ≤ 55				
			Case	Control	OR (95% CI)	*p*	FDR-*p*	Case	Control	OR (95% CI)	*p*	FDR-*p*
rs738409	Co-dominant	CC	72	90	1			82	90	1		
		CG	122	106	1.53(1.01–2.32)	**0.046**	**0.046**	128	134	1.04(0.71–1.53)	0.843	1.124
		GG	36	29	1.62(0.89–2.94)	0.114	0.227	43	33	1.42(0.82–2.44)	0.212	0.212
	Dominant	CC	72	90	1			82	90	1		
		CG + GG	158	135	1.55(1.04–2.30)	**0.030**	**0.030**	171	167	1.11(0.77–1.61)	0.565	0.754
	Recessive	CC + CG	194	196	1			210	224	1		
		GG	36	29	1.27(0.74–2.19)	0.390	1.599	43	33	1.38(0.84–2.26)	0.198	0.198
	Log-additive	–	–	–	1.33(1.00–1.76)	**0.047**	0.063	–	–	1.16(0.89–1.50)	0.276	0.368
rs3747207	Co-dominant	GG	72	90	1			84	91	1		
		GA	126	108	1.57(1.04–2.37)	**0.034**	**0.045**	128	140	0.98(0.67–1.44)	0.926	0.926
		AA	33	28	1.50(0.82–2.75)	0.192	0.192	41	30	1.47(0.84–2.57)	0.178	0.237
	Dominant	GG	72	90	1			84	91	1		
		GA + AA	159	136	1.55(1.05–2.30)	**0.029**	**0.039**	169	170	1.07(0.74–1.54)	0.726	0.726
	Recessive	GG + GA	198	198	1			212	231	1		
		AA	33	28	1.16(0.66–2.03)	0.605	0.605	41	30	1.48(0.89–2.47)	0.128	0.170
	Log-additive	–	–	–	1.31(0.98–1.74)	0.066	0.066	–	–	1.15(0.88–1.50)	0.294	0.294
rs4823173	Co-dominant	GG	70	90	1			82	94	1		
		GA	129	109	1.62(1.07–2.45)	**0.023**	**0.046**	127	133	1.09(0.74–1.60)	0.673	2.692
		AA	31	26	1.62(0.86–3.00)	0.137	0.183	44	30	1.66(0.96–2.88)	0.072	0.289
	Dominant	GG	70	90	1			82	94	1		
		GA + AA	160	135	1.62(1.09–2.40)	**0.018**	**0.036**	171	163	1.19(0.83–1.72)	0.348	1.394
	Recessive	GG + GA	199	199	1			209	227	1		
		AA	31	26	1.21(0.68–2.15)	0.515	1.030	44	30	1.58(0.96–2.61)	0.074	0.297
	Log-additive	–	–	–	1.36(1.02–1.82)	**0.040**	0.079	–	–	1.24(0.95–1.61)	0.109	0.435
rs2896019	Co-dominant	TT	68	92	1			82	94	1		
		TG	129	105	1.78(1.17–2.70)	**0.007**	**0.028**	128	136	1.07(0.73–1.57)	0.719	1.437
		GG	34	29	1.67(0.91–3.05)	0.098	0.391	42	31	1.54(0.89–2.68)	0.124	0.249
	Dominant	TT	68	92	1			82	94	1		
		TG + GG	163	134	1.75(1.18–2.61)	**0.006**	**0.023**	170	167	1.16(0.80–1.67)	0.428	0.855
	Recessive	TT + TG	197	197	1			210	230	1		
		GG	34	29	1.19(0.69–2.06)	0.539	0.719	42	31	1.48(0.89–2.44)	0.127	0.254
	Log-additive	–	–	–	1.40(1.05–1.86)	**0.022**	0.086	–	–	1.20(0.92–1.60)	0.172	0.344

*HCC* hepatocellular carcinoma; *SNP* single nucleotide polymorphism; *OR* odds ratio; *95% CI* 95% confidence interval; *FDR* false discovery rate

*p*-value was calculated by logistic regression analysis with adjustments for age, gender and smoking

Bold values indicate statistical significance

### Correlation between *PNPLA3* gene polymorphisms and serum tumor markers

Additionally, we also investigated the relationship between *PNPLA3* gene polymorphisms and serum tumor markers (Table 5). The results revealed that four candidate SNPs were not correlated with the levels of CEA, CA 50, CA 125, and CA 199 in HCC patients. However, rs738409 was significantly related to the levels of AFP in HCC patients (*p* = 0.007), and HCC patients with the GG genotype had a higher level of AFP.

**Table 5:** Association of *PNPLA3* gene polymorphisms with serum tumor marker levels

SNP_ID	Genotype	CEA (ng/mL)		AFP (ng/mL)		CA 50 (U/mL)		CA 125 (U/mL)		CA 199 (U/mL)	
		*n*	Mean ± SE	*n*	Mean ± SE	*n*	Mean ± SE	*n*	Mean ± SE	*n*	Mean ± SE
rs738409	CC	62	14.40 ± 0.80	49	41.50 ± 10.64	62	8.32 ± 1.34	47	143.46 ± 35.02	59	54.11 ± 10.36
	CG	98	15.54 ± 1.13	81	178.10 ± 103.35	78	7.54 ± 0.97	72	89.20 ± 17.13	92	51.50 ± 8.13
	GG	28	11.86 ± 1.48	21	1237.33 ± 790.25	20	10.30 ± 2.94	23	142.37 ± 41.92	28	62.71 ± 20.45
	*p*		0.391		**0.007**		0.543		0.245		0.826
rs3747207	GG	64	14.12 ± 0.80	49	41.50 ± 10.64	62	8.32 ± 1.34	49	138.79 ± 33.73	61	53.70 ± 10.03
	GA	99	15.87 ± 1.73	83	318.43 ± 174.55	79	7.84 ± 1.01	73	88.15 ± 16.92	93	52.06 ± 8.06
	AA	26	12.37 ± 1.55	20	699.20 ± 608.41	20	10.30 ± 2.94	21	153.15 ± 45.27	26	64.34 ± 22.00
	*p*		0.440		0.256		0.629		0.218		0.801
rs4823173	GG	61	14.36 ± 0.82	48	42.77 ± 10.83	61	8.45 ± 1.36	47	157.30 ± 36.60	59	54.53 ± 10.35
	GA	98	14.59 ± 1.13	82	321.99 ± 176.66	78	7.51 ± 0.97	71	81.00 ± 15.31	91	51.55 ± 8.22
	AA	29	11.86 ± 1.43	21	666.08 ± 579.66	21	9.93 ± 2.83	24	137.30 ± 40.35	29	61.33 ± 19.78
	*p*		0.373		0.284		0.598		0.086		0.86
rs2896019	TT	59	14.21 ± 0.85	46	95.18 ± 57.40	59	8.66 ± 1.40	46	158.12 ± 37.43	58	54.62 ± 10.53
	TG	101	16.08 ± 1.70	85	284.68 ± 168.21	81	7.70 ± 0.99	73	80.97 ± 14.89	93	52.18 ± 8.060
	GG	29	11.52 ± 1.33	21	660.54 ± 579.94	21	9.82 ± 2.84	24	136.13 ± 40.52	29	61.02 ± 19.80
	*p*		0.250		0.377		0.663		0.082		0.884

*SNP* single nucleotide polymorphism; *CEA* carcino-embryonic antigen; *AFP* alpha-fetoprotein; *CA 50* carbohydrate antigen 50; *CA 125* carbohydrate antigen 125; *CA 199* carbohydrate antigen 199; *n* number; *SE* standard error

Bold values indicate statistical significance

*p*-values were calculated using *χ*^2^ tests

### Linkage disequilibrium (LD) and haplotype analysis

**Fig. 1:**
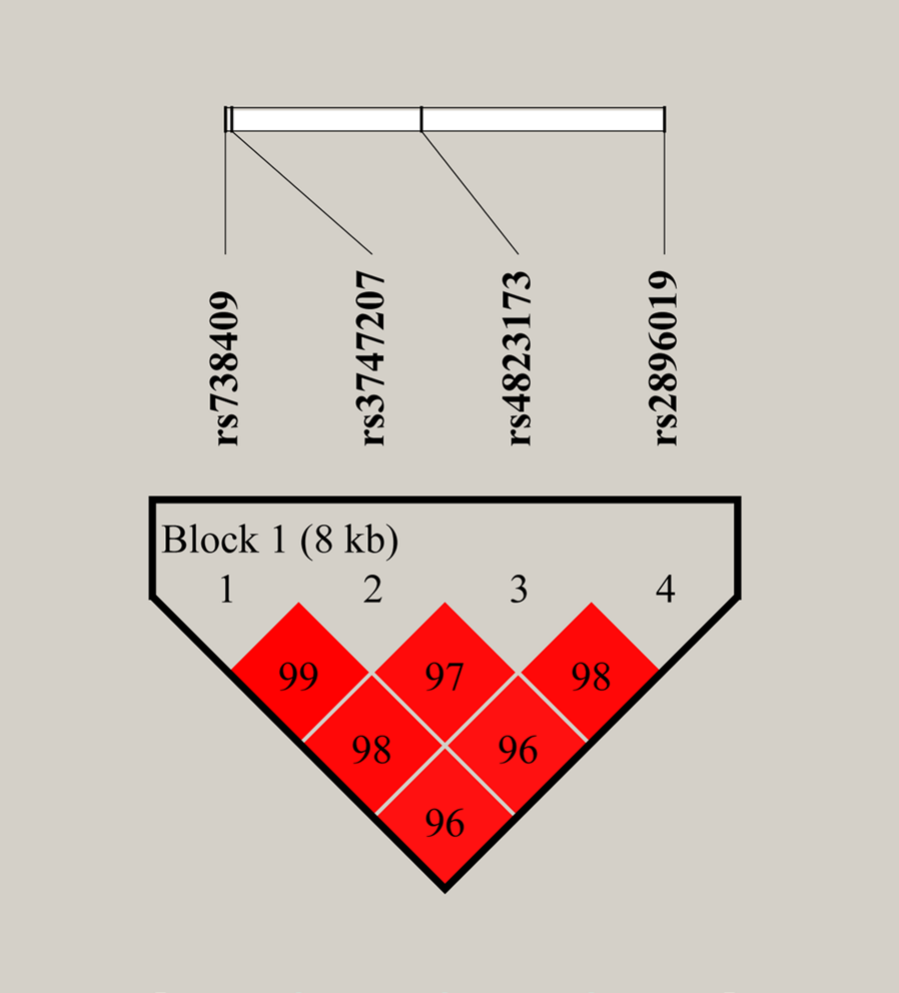
LD analysis of *PNPLA3* SNPs. Red squares represent statistically significant associations between SNPs, as measured by D’. Darker red indicates higher D’. LD: linkage disequilibrium; SNP: single nucleotide polymorphism

LD analysis of *PNPLA3* SNPs (rs738409, rs3747207, rs4823173, and rs2896019) was carried out using Haploview software. The results showed that there were linkages between SNPs, and one LD block was obtained, namely rs738409-rs3747207-rs4823173-rs2896019 (Fig. 1). At the same time, haplotype analyses of *PNPLA3* SNPs were performed, and haplotypes with frequencies < 0.03 were ignored. As shown in Table 6, one haplotype, rs738409 (G)-rs3747207 (A)-rs4823173 (A)-rs2896019 (G), was detected. We found that the GAAG haplotype was significantly associated with increased HCC susceptibility (OR = 1.25, 95% CI = 1.03–1.53, *p* = 0.023). After FDR correction, this haplotype was still significantly associated with increased HCC susceptibility (FDR-*p* = 0.046).

**Table 6:** Haplotype frequencies of *PNPLA3* polymorphisms and their association with HCC susceptibility

SNP-ID	Haplotype	Fre-HCC	Fre-control	OR (95% CI)	*p*	FDR-*p*
rs738409-rs3747207-rs4823173-rs2896019	CGGT	0.564	0.613	1		
	GAAG	0.406	0.360	1.25(1.03–1.53)	**0.023**	**0.046**

*HCC* hepatocellular carcinoma; *SNP* single nucleotide polymorphism; *Fre* frequency; *OR* odds ratio; *95% CI* 95% confidence interval; *FDR* false discovery rate

Adjusted OR (95% CI) was calculated by logistic regression analysis

Bold values indicate statistical significance

### MDR analysis

**Fig. 2:**
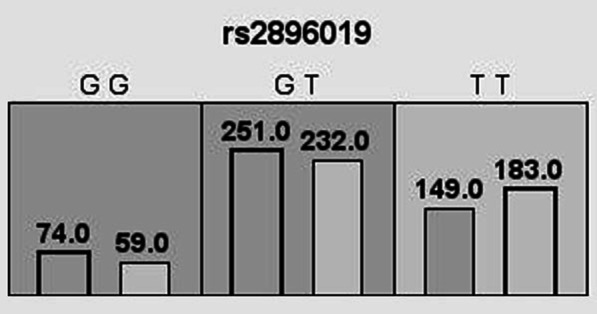
Genotype distribution of *PNPLA3* rs2896019 in cases and controls based on MDR analysis. For each genotype, the number of cases is displayed in the histogram on the left of each cell, while the number of controls is displayed on the right. Darker shadows indicate higher HCC susceptibility. MDR: multifactor dimensionality reduction; HCC: hepatocellular carcinoma

SNP-SNP interactions were analyzed using MDR software (Table 7). As a result, we found that the best single-locus prediction model was rs2896019, and HCC patients carrying rs2896019 GG and GT genotypes showed a testing balanced accuracy of 0.536 and a CVC of 10/10 for predicting HCC susceptibility. The rs2896019 GG and GC genotypes had 1.25-fold and 1.08-fold increased susceptibility to HCC, respectively (CVC = 10/10, testing balanced accuracy = 0.536, OR = 1.37, 95% CI = 1.05–1.79, *p* = 0.021; Fig. 2). Moreover, the best multi-locus prediction model was the four-locus model (the combination of rs738409, rs3747207, rs4823173, and rs2896019) (CVC = 10/10, testing balanced accuracy = 0.532, OR = 1.48, 95% CI = 1.13–1.93, *p* = 0.004).

**Table 7:** SNP-SNP interaction models of candidate SNPs analyzed by the MDR method

Model	Training Bal. Acc	Testing Bal. Acc	CVC	OR (95% CI)	*p*
rs2896019	0.536	0.536	10/10	1.37 (1.05–1.79)	**0.021**
rs738409,rs4823173	0.539	0.527	6/10	1.39(1.06–1.81)	**0.015**
rs738409,rs4823173,rs2896019	0.523	0.523	9/10	1.45(1.11–1.89)	**0.007**
rs738409,rs3747207,rs4823173,rs2896019	0.546	0.532	10/10	1.48(1.13–1.93)	**0.004**

*SNP* single nucleotide polymorphism; *MDR* multifactor dimensionality reduction; *Bal. Acc.* balanced accuracy; *CVC* cross-validation consistency; *OR* odds ratio; *95% CI* 95% confidence interval

Bold values indicate statistical significance

*p*-values were calculated using *χ*^2^ tests

### FPRP analysis

FPRP analysis was used to verify the reliability of the observed associations between *PNPLA3* SNPs and HCC susceptibility (Table 8). The FPRP values were less than 0.2, indicating the associations were noteworthy. When the prior probability was 0.25, the significant associations between all SNPs and increased HCC susceptibility were notable in all genetic models, except for rs738409 CG versus CC model in the overall and stratified analyses (age > 55). When the prior probability was 0.1, the significant associations of rs4823173 and rs2896019 with increased susceptibility to HCC were more notable in different genetic models: rs4823173 A versus G (FPRF = 0.132, power = 0.975) and log-additive (FPRP = 0.098, power = 0.948) model in the overall analysis, rs2896019 G versus T (FPRF = 0.132, power = 0.975), TG versus TT (FPRF = 0.109, power = 0.856), TG + GG versus TT (FPRF = 0.198, power = 0.727), and log-additive (FPRF = 0.098, power = 0.948) model in the overall analysis, and rs2896019 TG + GG versus TT (FPRF = 0.195, power = 0.225) model in stratified analysis (age > 55). Since the relationship between four SNPs and HCC susceptibility might not be affected by gender and smoking status, FPRP analysis was not performed in subgroups stratified by gender and smoking.

**Table 8:** FPRP analysis of significant findings

SNP_ID	Genotype	Crude OR (95% CI)	*p*	Statistical power	Prior probability				
					0.25	0.1	0.01	0.001	0.0001
rs738409	*Overall analysis*								
	G versus C	1.21(1.00–1.45)	0.039	0.990	**0.106**	0.261	0.796	0.975	0.997
	GG versus CC	1.49(1.00–2.22)	0.045	0.500	0.213	0.448	0.899	0.989	0.999
	Log-additive	1.22(1.01–1.48)	0.044	0.982	**0.118**	0.286	0.815	0.978	0.998
	*Age > 55*								
	CG versus CC	1.53(1.01–2.32)	0.045	0.163	0.227	0.468	0.906	0.990	0.999
	CG + GG versus CC	1.55(1.04–2.30)	0.030	0.435	**0.169**	0.379	0.870	0.985	0.999
	Log-additive	1.33(1.00–1.76)	0.046	0.800	**0.147**	0.341	0.851	0.983	0.998
rs3747207	*Age > 55*								
	GA versus GG	1.57(1.04–2.37)	0.032	0.414	**0.187**	0.409	0.884	0.987	0.999
	GA + AA versus GG	1.55(1.05–2.30)	0.030	0.435	**0.169**	0.379	0.870	0.985	0.999
rs4823173	*Overall analysis*								
	A versus G	1.25(1.04–1.50)	0.016	0.975	**0.048**	**0.132**	0.625	0.944	0.994
	AA versus GG	1.63(1.08–2.46)	0.020	0.346	**0.148**	0.342	0.851	0.983	0.998
	GA + AA versus GG	1.35(1.04–1.77)	0.030	0.777	**0.103**	0.257	0.792	0.975	0.997
	Log-additive	1.28(1.06–1.55)	0.011	0.948	**0.035**	**0.098**	0.545	0.924	0.992
	*Age > 55*								
	GA versus GG	1.62(1.07–2.45)	0.022	0.358	**0.157**	0.359	0.860	0.984	0.998
	GA + AA versus GG	1.62(1.09–2.40)	0.016	0.351	**0.121**	0.293	0.820	0.979	0.998
	Log-additive	1.36(1.02–1.82)	0.039	0.745	**0.134**	0.318	0.837	0.981	0.998
rs2896019	*overall analysis*								
	G versus T	1.25(1.04–1.50)	0.016	0.975	**0.048**	**0.132**	0.625	0.944	0.994
	TG versus TT	1.33(1.01–1.76)	0.012	0.856	**0.039**	**0.109**	0.574	0.932	0.993
	GG versus TT	1.59(1.06–2.39)	0.026	0.390	**0.165**	0.373	0.867	0.985	0.998
	TG + GG versus TT	1.38(1.06–1.81)	0.020	0.727	**0.076**	**0.198**	0.731	0.965	0.996
	Log-additive	1.28(1.06–1.55)	0.011	0.948	**0.035**	**0.098**	0.545	0.924	0.992
	*Age > 55*								
	TG versus TT	1.78(1.17–2.70)	0.007	0.210	**0.087**	0.222	0.759	0.969	0.997
	TG + GG versus TT	1.75(1.18–2.61)	0.006	0.225	**0.075**	**0.195**	0.728	0.964	0.996
	Log-additive	1.40(1.05–1.86)	0.020	0.683	**0.082**	0.211	0.746	0.967	0.997

*FPRP* False-positive report probability; *SNP* single nucleotide polymorphism; *OR* odds ratio; *CI* confidence interval

FPRP values were less than 0.2, indicating that the positive results were noteworthy

Bold values represent noteworthy findings

## Discussion

HCC has become a public health problem worldwide, especially in China, which has the highest morbidity and lethality rate. And about 50% of newly-diagnosed HCC cases and HCC deaths in the world occur in China [17, 18]. Since SNPs are considered as one of the major risk factors for HCC, it is of great significance to study the association between SNPs and HCC susceptibility [19, 20]. In our study, the association of *PNPLA3* SNPs with HCC susceptibility was analyzed in 484 HCC patients and 487 healthy controls, and the results showed that rs2896019 was significantly associated with increased HCC susceptibility. Our research contributes to the understanding of the pathogenesis of HCC and provides a new method for the treatment of HCC.

To date, researches on *PNPLA3* gene polymorphisms have mainly focused on rs738409 and its relationship with NAFLD risk. The study by Hikmet Akkiz et al. has shown that rs738409 markedly increases the risk of NAFLD in the Turkish population in an unadjusted regression model [21]. Besides, the study by Daniel F Mazo et al. has also discovered that Brazilian subjects with the rs738409-GG genotype have a 3.29-fold increased risk of NAFLD [22]. Meanwhile, a study using a meta-analysis approach has highlighted that people with rs738409 CG and GG genotypes have a 19% and 105% likelihood of developing NAFLD, respectively [11]. Furthermore, one study has revealed the association between *PNPLA3* rs738409 and HCC risk and demonstrated that rs738409 is an independent predictor of HCC occurrence [23]. However, the above study was conducted in white Italian patients, and racial differences may lead to the inconsistency between our results and those of the above study. We also explored the association of rs3747207, rs4823173 and rs2896019 with HCC susceptibility. One study has also revealed the significant correlation of *PNPLA3* rs4823173 and rs2896019 with HCC susceptibility [24]. Notably, although no association was observed between the alleles and genotypes of rs4823173 and HCC susceptibility, our study demonstrated that the rs2896019 G allele and GG genotype were obviously associated with increased HCC susceptibility. However, so far, only one study has reported that rs3747207 is linked to NAFLD [25]. No significant association of rs3747207 with HCC susceptibility was observed in our study, which might be related to the discrepancy between diseases.

Given multiple factors for HCC occurrence, our study explored the relationship between *PNPLA3* SNPs and HCC susceptibility in terms of age, gender and smoking status. First, in terms of age, the mean age of HCC cases in our study was 55 years, and people aged more than 55 years had an increased risk of HCC. Specifically, subjects aged more than 55 years with rs738409 CG, rs3747207 GA, rs4823173 GA, and rs2896019 TG were more likely to develop HCC. This may be due to the decline of the body’s immunity with age, thus leading to the increased risk of HCC. Second, as for gender, studies around the world have shown that HCC is a male-oriented malignancy [26]. In most countries, men are 2 to 4 times, or even 3 to 5 times more likely to suffer from HCC than women [27–29]. The male to female ratio of HCC incidence in this study was 3.35:1, which is basically consistent with the international gender trend of HCC incidence. This difference in gender distribution is thought to be closely related to hormone levels in men and their unhealthy lifestyles like overworking, staying up late and excessive drinking. Third, in terms of smoking status, studies have shown that smoking is a minor risk factor for HCC [30, 31]. According to reports, smoking can increase the morbidity and mortality of HCC [32]. Our study showed that there was no significant difference in the distribution of smoking between cases and controls, indicating that smoking failed to affect the association of selected SNPs with the occurrence of HCC. This contradiction may be due to the randomness of sample selection.

Considering that HCC is a complex polygenic disease, studies on SNP-SNP interactions may help identify risk factors for HCC. Of note, MDR is an effective method for detecting SNP-SNP interactions in case–control studies. In this study, MDR analysis was used to analyze the interactions between four candidate SNPs, and the results showed that the best single-locus model for predicting HCC susceptibility was the model (rs2896019). This result was consistent with that of the overall analysis, that is, rs2896019 was significantly associated with increased susceptibility to HCC. The best multi-locus prediction model was the four-locus model, the combination of rs738409, rs3747207, rs4823173, and rs2896019, which might further support the influence of SNP-SNP interactions on HCC susceptibility. However, the complex interactions between *PNPLA3* SNPs in the progression of HCC remain to be further investigated.

In conclusion, rs2896019 increased HCC susceptibility. At the same time, FPRP analysis validated the reliability of the significant associations in our findings. However, the current study was designed to research only one gene, *PNPLA3*, and its SNPs. In the follow up studies, we will select multiple genes that share the same molecular pathway with *PNPLA3* and their corresponding SNPs to better investigate the relationship between genetic polymorphisms and HCC.

## Conclusions

This study revealed that *PNPLA3* rs2896019 was associated with an increased susceptibility to HCC, but what roles these *PNPLA3* SNPs play in the occurrence and development of HCC remains to be further studied.

## Supplementary Information

**Additional file 1:** Primer sequence of four *PNPLA3* SNPs and the relationship between *PNPLA3* polymorphisms and HCC susceptibility stratified by gender and smoking.

## Data Availability

The data that support our findings are available from the corresponding author upon reasonable request.
