# Supplementary material for: Contribution of PNPLA3 gene polymorphisms to hepatocellular carcinoma susceptibility in the Chinese Han population
Source: BMC Med Genomics. 2022 Nov 29;15:248. doi: 10.1186/s12920-022-01394-7 (PMC9706882; doi:10.1186/s12920-022-01394-7)
Supplement: Supplementary file 1 — Additional file 1: Primer sequence of four PNPLA3 SNPs and the relationship between PNPLA3 polymorphisms and HCC susceptibility stratified by gender and smoking. [file 12920_2022_1394_MOESM1_ESM.docx]

**Table S1.** **The** **primers of the selected SNPs.**

| **SNP_ID** | **1st-PCRP** | **2nd-PCRP** | **UEP_SEQ** | **Director** |
| --- | --- | --- | --- | --- |
| rs738409 | ACGTTGGATGAGAGAAAGCCGACTTACCAC | ACGTTGGATGTCACAGGCCTTGGTATGTTC | GGTATGTTCCTGCTTCAT | F |
| rs3747207 | ACGTTGGATGAAGTGTGCTCACACATCTCC | ACGTTGGATGTCTGTCTGAAAGGCAGTGAG | ACAGACCCTGAGGTGCC | R |
| rs4823173 | ACGTTGGATGACTCCTCCATCCACATATCG | ACGTTGGATGGCACTTAAGCACTAACCCAG | gaaaCCAGAGCTTCAGACAGT | F |
| rs2896019 | ACGTTGGATGTTGGACCATAGCACTGTCTC | ACGTTGGATGACCCACAATTTTGAACCTCC | cccaTCCATCGAATGGTGCTGTA | F |

SNP: single nucleotide polymorphism; PCRP: polymerase chain reaction primer; UEP: unextended mini sequencing primer; SEQ: sequence.

**Table S2. Association between *PNPLA3* polymorphisms and HCC susceptibility stratified by gender.**

| **SNP_ID** | **Model** | **Genotype** | **Male** | | | | | **Female** | | | | |
| --- | --- | --- | --- | --- | --- | --- | --- | --- | --- | --- | --- | --- |
|  |  |  | **Case** | **Control** | **OR (95% CI)** | ***p*** | **FDR-*p*** | **Case** | **Control** | **OR (95% CI)** | ***p*** | **FDR-*p*** |
| rs738409 | Co-dominant | CC | 125 | 138 | 1 |  |  | 29 | 42 | 1 |  |  |
|  |  | CG | 192 | 197 | 1.06(0.78-1.46) | 0.701 | 0.934 | 58 | 43 | 2.33(1.21-4.50) | **0.012** | **0.024** |
|  |  | GG | 59 | 40 | 1.67(1.04-2.67) | **0.033** | **0.333** | 20 | 22 | 1.07(0.47-2.2.42) | 0.877 | 1.169 |
|  | Dominant | CC | 125 | 138 | 1 |  |  | 29 | 42 | 1 |  |  |
|  |  | CG+GG | 251 | 237 | 1.16(0.86-1.57) | 0.322 | 0.429 | 78 | 65 | 1.83(1.00-3.35) | **0.049** | 0.099 |
|  | Recessive | CC+CG | 317 | 335 | 1 |  |  | 87 | 85 | 1 |  |  |
|  |  | GG | 59 | 40 | 1.61(1.04-2.48) | **0.031** | **0.042** | 20 | 22 | 0.67(0.32-1.40) | 0.286 | 0.571 |
|  | Log-additive | — | — | — | 1.23(0.99-1.53) | 0.068 | 0.068 | — | — | 1.16(0.78-1.72) | 0.467 | 0.632 |
| rs3747207 | Co-dominant | GG | 127 | 140 | 1 |  |  | 29 | 41 | 1 |  |  |
|  |  | GA | 194 | 200 | 1.06(0.77-1.44) | 0.732 | 0.732 | 60 | 48 | 2.03(1.07-3.88) | **0.031** | **0.031** |
|  |  | AA | 55 | 35 | 1.76(1.08-2.88) | **0.023** | **0.030** | 19 | 23 | 1.02(0.45-2.30) | 0.969 | 0.969 |
|  | Dominant | GG | 127 | 140 | 1 |  |  | 29 | 41 | 1 |  |  |
|  |  | GA+AA | 249 | 235 | 1.16(0.86-1.57) | 0.330 | 0.330 | 79 | 71 | 1.66(0.91-3.01) | 0.098 | 0.098 |
|  | Recessive | GG+GA | 321 | 340 | 1 |  |  | 89 | 89 | 1 |  |  |
|  |  | AA | 55 | 35 | 1.71(1.09-2.69) | **0.020** | **0.041** | 19 | 23 | 0.68(0.33-1.39) | 0.289 | 0.385 |
|  | Log-additive | — | — | — | 1.24(0.99-1.55) | 0.059 | 0.079 | — | — | 1.11(0.75-1.64) | 0.605 | 0.605 |
| rs4823173 | Co-dominant | GG | 123 | 142 | 1 |  |  | 29 | 42 | 1 |  |  |
|  |  | GA | 198 | 195 | 1.16(0.85-1.59) | 0.341 | 1.356 | 58 | 47 | 2.05(1.07-3.91) | **0.029** | **0.039** |
|  |  | AA | 55 | 37 | 1.77(1.09-2.87) | **0.021** | **0.042** | 20 | 19 | 1.31(0.57-3.02) | 0.524 | 2.098 |
|  | Dominant | GG | 123 | 142 | 1 |  |  | 29 | 42 | 1 |  |  |
|  |  | GA+AA | 253 | 232 | 1.26(0.93-1.70) | 0.133 | 0.267 | 78 | 66 | 1.81(0.99-3.31) | 0.054 | 0.072 |
|  | Recessive | GG+GA | 321 | 337 | 1 |  |  | 87 | 89 | 1 |  |  |
|  |  | AA | 55 | 37 | 1.62(1.03-2.53) | **0.035** | **0.035** | 20 | 19 | 0.87(0.41-1.83) | 0.711 | 0.711 |
|  | Log-additive | — | — | — | 1.28(1.02-1.60) | **0.030** | 0.060 | — | — | 1.26(0.84-1.89) | 0.267 | 1.067 |
| rs2896019 | Co-dominant | TT | 124 | 144 | 1 |  |  | 26 | 42 | 1 |  |  |
|  |  | TG | 195 | 194 | 1.15(0.84-1.58) | 0.369 | 0.737 | 62 | 47 | 2.48(1.29-4.78) | **0.007** | **0.027** |
|  |  | GG | 57 | 37 | 1.85(1.15-3.00) | **0.012** | **0.048** | 19 | 23 | 1.11(0.49-2.56) | 0.792 | 1.056 |
|  | Dominant | TT | 124 | 144 | 1 |  |  | 26 | 42 | 1 |  |  |
|  |  | TG+GG | 252 | 231 | 1.26(0.94-1.71) | 0.126 | 0.502 | 81 | 70 | 1.97(1.07-3.62) | **0.030** | 0.118 |
|  | Recessive | TT+TG | 319 | 338 | 1 |  |  | 88 | 89 | 1 |  |  |
|  |  | GG | 57 | 37 | 1.70(1.09-2.66) | **0.019** | 0.076 | 19 | 23 | 0.66(0.32-1.35) | 0.254 | 1.016 |
|  | Log-additive | — | — | — | 1.30(1.04-1.62) | **0.021** | 0.084 | — | — | 1.18(0.79-1.76) | 0.409 | 0.818 |

HCC: hepatocellular carcinoma; SNP: single nucleotide polymorphism; OR: odds ratio; 95% CI: 95% confidence interval; FDR: false discovery rate.

*p*-value was calculated by logistic regression analysis with adjustments for age, gender and smoking.

Bold values indicate statistical significance.

**Table S3. Association between *PNPLA3* polymorphisms and HCC susceptibility stratified by smoking.**

| **SNP_ID** | **Model** | **Genotype** | **Smoking** | | | | | **Non-smoking** | | | | |
| --- | --- | --- | --- | --- | --- | --- | --- | --- | --- | --- | --- | --- |
|  |  |  | **Case** | **Control** | **OR (95% CI)** | ***p*** | **FDR-*p*** | **Case** | **Control** | **OR (95% CI)** | ***p*** | **FDR-*p*** |
| rs738409 | Co-dominant | CC | 81 | 83 | 1 |  |  | 73 | 97 | 1 |  |  |
|  |  | CG | 122 | 125 | 0.95(0.63-1.43) | 0.809 | 1.079 | 128 | 115 | 1.60(1.07-2.38) | **0.021** | **0.028** |
|  |  | GG | 35 | 24 | 1.77(0.95-3.30) | 0.071 | 0.071 | 44 | 38 | 1.41(0.83-2.42) | 0.205 | 0.273 |
|  | Dominant | CC | 81 | 83 | 1 |  |  | 73 | 97 | 1 |  |  |
|  |  | CG+GG | 157 | 149 | 1.07(0.73-1.59) | 0.719 | 0.719 | 172 | 153 | 1.55(1.06-2.26) | **0.022** | **0.030** |
|  | Recessive | CC+CG | 203 | 208 | 1 |  |  | 201 | 212 | 1 |  |  |
|  |  | GG | 35 | 24 | 1.83(1.03-3.24) | **0.039** | 0.052 | 44 | 28 | 1.08(0.67-1.75) | 0.761 | 1.523 |
|  | Log-additive | — | — | — | 1.21(0.91-1.61) | 0.183 | 0.183 | — | — | 1.26(0.97-1.63) | 0.081 | 0.108 |
| rs3747207 | Co-dominant | GG | 82 | 84 | 1 |  |  | 74 | 97 | 1 |  |  |
|  |  | GA | 123 | 129 | 0.93(0.62-1.39) | 0.723 | 1.447 | 131 | 119 | 1.55(1.05-2.29) | **0.029** | **0.029** |
|  |  | AA | 33 | 21 | 2.13(1.12-4.06) | **0.022** | **0.029** | 41 | 37 | 1.20(0.69-2.08) | 0.517 | 0.517 |
|  | Dominant | GG | 82 | 84 | 1 |  |  | 74 | 97 | 1 |  |  |
|  |  | GA+AA | 156 | 150 | 1.08(0.73-1.60) | 0.692 | 0.922 | 172 | 156 | 1.46(1.01-2.12) | **0.047** | **0.047** |
|  | Recessive | GG+GA | 205 | 213 | 1 |  |  | 205 | 216 | 1 |  |  |
|  |  | AA | 33 | 21 | 2.22(1.22-4.05) | **0.009** | **0.036** | 41 | 37 | 0.93(0.56-1.53) | 0.775 | 1.033 |
|  | Log-additive | — | — | — | 1.27(0.95-1.69) | 0.101 | 0.135 | — | — | 1.18(0.91-1.54) | 0.209 | 0.209 |
| rs4823173 | Co-dominant | GG | 81 | 87 | 1 |  |  | 71 | 97 | 1 |  |  |
|  |  | GA | 123 | 126 | 0.99(0.66-1.48) | 0.972 | 0.972 | 133 | 116 | 1.70(1.14-2.53) | **0.009** | **0.035** |
|  |  | AA | 34 | 21 | 2.19(1.14-4.18) | **0.018** | 0.072 | 41 | 35 | 1.42(0.82-2.47) | 0.211 | 0.422 |
|  | Dominant | GG | 81 | 87 | 1 |  |  | 71 | 97 | 1 |  |  |
|  |  | GA+AA | 157 | 147 | 1.15(0.78-1.69) | 0.493 | 0.985 | 174 | 151 | 1.63(1.12-2.38) | **0.011** | **0.022** |
|  | Recessive | GG+GA | 204 | 213 | 1 |  |  | 204 | 213 | 1 |  |  |
|  |  | AA | 34 | 21 | 2.19(1.20-4.01) | **0.011** | **0.021** | 41 | 35 | 1.04(0.63-1.71) | 0.873 | 0.873 |
|  | Log-additive | — | — | — | 1.30(0.98-1.74) | 0.070 | 0.282 | — | — | 1.29(0.99-1.67) | 0.061 | 0.123 |
| rs2896019 | Co-dominant | TT | 79 | 88 | 1 |  |  | 71 | 98 | 1 |  |  |
|  |  | TG | 125 | 123 | 1.08(0.72-1.61) | 0.713 | 2.852 | 132 | 118 | 1.67(1.13-2.49) | **0.011** | **0.022** |
|  |  | GG | 34 | 23 | 1.89(1.00-3.57) | 0.050 | 0.100 | 42 | 37 | 1.51(0.88-2.59) | 0.133 | 0.533 |
|  | Dominant | TT | 79 | 88 | 1 |  |  | 71 | 98 | 1 |  |  |
|  |  | TG+GG | 159 | 146 | 1.20(0.81-1.76) | 0.367 | 1.467 | 174 | 155 | 1.63(1.12-2.38) | **0.011** | **0.043** |
|  | Recessive | TT+TG | 204 | 211 | 1 |  |  | 203 | 216 | 1 |  |  |
|  |  | GG | 34 | 23 | 1.81(1.00-3.26) | **0.049** | **0.049** | 42 | 37 | 1.12(0.69-1.81) | 0.659 | 2.638 |
|  | Log-additive | — | — | — | 1.28(0.96-1.70) | 0.097 | 0.194 | — | — | 1.30(1.01-1.69) | **0.045** | 0.180 |

HCC: hepatocellular carcinoma; SNP: single nucleotide polymorphism; OR: odds ratio; 95% CI: 95% confidence interval; FDR: false discovery rate.

*p*-value was calculated by logistic regression analysis with adjustments for age, gender and smoking.

Bold values indicate statistical significance.
